# Spontaneous Coronary Artery Dissection in a Lactating Mother Three Years Postpartum: A Rare Case Report

**DOI:** 10.7759/cureus.96330

**Published:** 2025-11-07

**Authors:** Charles O Poluyi, Esther F Duodu, Elle Le, Omar A Oudit, Shahrokh E Rafii

**Affiliations:** 1 Internal Medicine, Brookdale University Hospital and Medical Center, Brooklyn, USA; 2 Internal Medicine, American University of Antigua, Antigua, ATG; 3 Biochemistry, City College of New York, Manhattan, USA; 4 Internal Medicine and Biochemistry, Touro College of Osteopathic Medicine, Manhattan, USA; 5 Cardiology, Brookdale University Hospital and Medical Center, Brooklyn, USA

**Keywords:** acute coronary syndrome, breastfeeding-associated scad, coronary tortuosity, delayed postpartum scad, prolonged lactation, women’s cardiovascular health

## Abstract

Spontaneous coronary artery dissection (SCAD) is a rare cause of acute coronary syndrome (ACS), typically seen in young women during or shortly after pregnancy. SCAD occurring beyond one year postpartum is uncommon, and no clear association has been established with prolonged lactation.

We present the case of a 41-year-old woman, actively breastfeeding her three-year-old child, with no traditional cardiovascular risk factors. She presented with ST-elevation myocardial infarction (STEMI). Coronary angiography revealed a sudden change in the calibre of the distal left anterior descending artery (LAD), followed by a long segment of narrowing and eventual subtotal occlusion. The clinical picture and angiographic findings were consistent with SCAD. She was managed conservatively and is undergoing further evaluation for fibromuscular dysplasia and other arteriopathies.

This case represents a rare, delayed postpartum presentation of SCAD in the context of prolonged lactation, with angiographic features of coronary tortuosity and subtotal occlusion of the distal LAD. It highlights the need to consider SCAD even years after childbirth and in patients with non-traditional risk factors.

## Introduction

Spontaneous coronary artery dissection (SCAD) is a spontaneous separation of the layers of the coronary artery wall, typically between the tunica intima and tunica media, or within the tunica media itself. This separation creates a false lumen, which can compress the true lumen, leading to myocardial ischaemia or infarction. The aetiology of SCAD can be grouped into atherosclerotic causes, plaque-related events leading to a dissection flap or intramural haematoma, or non-atherosclerotic causes. In this case, we review SCAD of non-atherosclerotic aetiology, which is the most common. It may be due to fibromuscular dysplasia (FMD), the peri-partum state, extreme stress, connective tissue disorders, or idiopathic mechanisms.

SCAD accounts for up to 35% of acute coronary syndrome (ACS) cases in women under 50 and is the leading cause of myocardial infarction associated with pregnancy [1]. SCAD is most strongly linked to the peripartum and early postpartum periods, while its occurrence years after delivery is uncommon. Hormonal and structural vascular changes during and after pregnancy are believed to contribute to arterial wall fragility, predisposing to SCAD. Importantly, SCAD is not limited to pregnancy-related cases; it can also occur in non-pregnant individuals, with several studies identifying predisposing factors such as hormonal therapy, emotional stress, and underlying arteriopathies, including FMD and connective tissue disorders [1-3]. Hormonal therapies, including oral contraceptives, have been implicated in altering vascular structure and may increase susceptibility to dissection. These findings underscore the need to consider SCAD in a broader clinical context, even outside pregnancy.

The relationship between prolonged lactation of up to three years and SCAD has not been previously characterised in the literature. Coronary artery tortuosity and distal vessel occlusions are emerging angiographic features in SCAD patients and reflect an underlying predisposition to arteriopathy. Research has identified that findings such as tortuosity are often associated with other vascular pathologies, like FMD, which is present in a significant number of SCAD cases [4,5]. FMD predisposes patients to arterial wall degradation, resulting in the formation of dissections and various arterial anomalies, including tortuosity [6]. Given the marked coronary artery tortuosity observed on angiography in the absence of atherosclerotic disease, FMD is the most likely underlying aetiology, consistent with previously reported associations between FMD and arterial tortuosity.

## Case presentation

A 41-year-old female presented to the hospital with the sudden onset of substernal chest pain. The pain initially improved before bedtime but recurred during sleep, awakening her from rest. It was non-radiating, with no associated symptoms, and worsened gradually over a few hours, resolving only after a loading dose of aspirin was administered by EMS. Before the onset of pain, the patient reported experiencing significant stress at home. She had no previous history of hypertension, diabetes, hyperlipidaemia, smoking, family history of coronary artery disease (CAD), or connective tissue disease but had a notable history of prolonged lactation, as she was still actively breastfeeding her three-year-old child.

**Figure 1:**
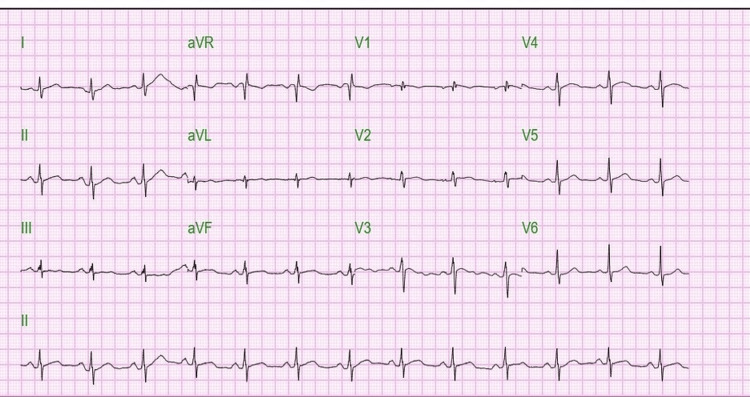
Initial EKG on presentation showing normal sinus rhythm (NSR) with no ST-T wave changes. aVR: Augmented Voltage Right arm; aVL: Augmented Voltage Left arm; aVF: Augmented Voltage Foot.

Initial ECG on presentation showed sinus rhythm with no ST-T wave changes (Figure 1), but high-sensitivity troponin was 143 pg/mL at admission (0 hours), increased to 796 pg/mL at 6 hours, and peaked at 9513 pg/mL at 36 hours (normal < 5-11.8) (Table 1). Initial vital signs were stable: BP 122/76 mmHg, HR 68 bpm, RR 16, SpO₂ 99% on room air, Temp 99 °F. Physical examination was notable for persistent melasma on the face since pregnancy. The patient was admitted for non-ST-elevation myocardial infarction (NSTEMI) management, with concern for Takotsubo cardiomyopathy due to preceding emotional stress.

**Table 1:** Troponin levels measured using a high-sensitivity assay. Troponin levels were measured using a high-sensitivity assay. The patient’s initial troponin was 143 pg/mL (reference range: <11.8 pg/mL) and peaked at 9513 pg/mL following recurrent chest pain and coronary angiography findings suggestive of spontaneous coronary artery dissection (SCAD).

Sampling time (hours)	Parameter	Patient value (pg/mL)	Reference range (pg/mL)
0 (Admission)	High-sensitivity troponin (hs-Tn)	143	<5-11.8
6	High-sensitivity troponin (hs-Tn)	796	<5-11.8
36	High-sensitivity troponin (hs-Tn)	9513	<5-11.8

**Figure 2:**
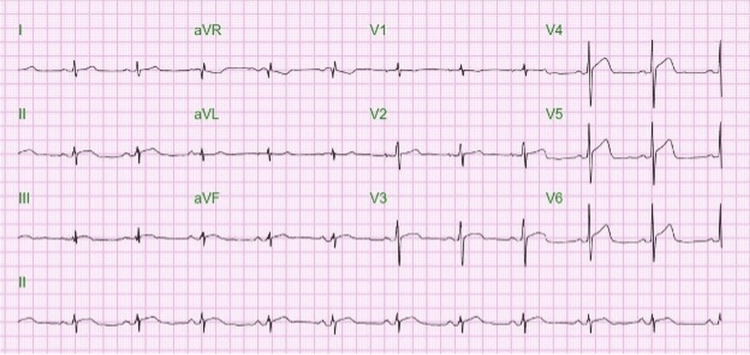
EKG at the time of chest pain prior to cardiac catheterization. aVR: Augmented Voltage Right arm; aVL: Augmented Voltage Left arm; aVF: Augmented Voltage Foot.

On day 2 of admission, the patient developed another episode of sudden, severe substernal chest pain associated with nausea, vomiting, diaphoresis, and radiation to the left arm. ECG showed ST elevation in leads V3-V6 without reciprocal changes (Figure 2). The patient was sent for emergent cardiac catheterisation.

**Figure 3:**
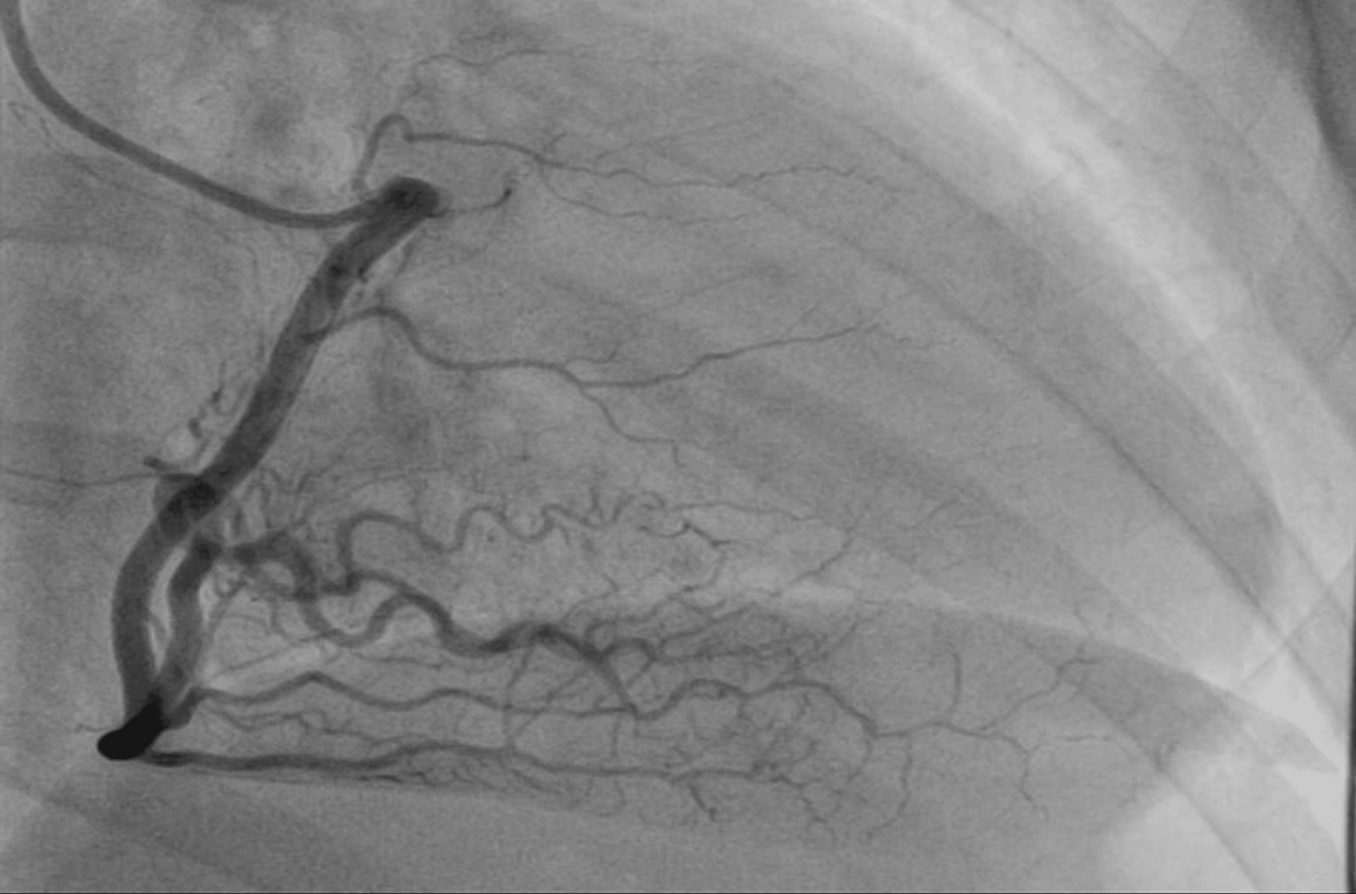
Coronary angiogram showing marked tortuosity of the right coronary artery.

**Figure 4:**
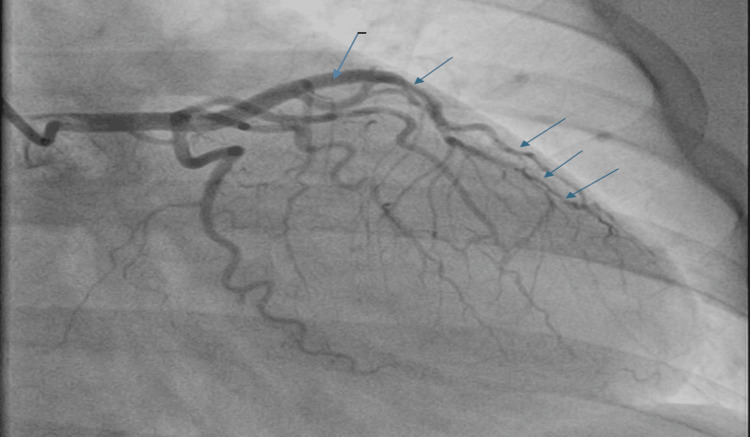
Coronary angiogram (RAO cranial view) demonstrating the left coronary system with culprit lesions (arrows). The angiogram shows a type 2 spontaneous coronary artery dissection (SCAD) involving the mid-left anterior descending (LAD) artery, characterized by long segmental narrowing with preserved distal flow (TIMI 3). RAO: Right anterior oblique.

**Figure 5:**
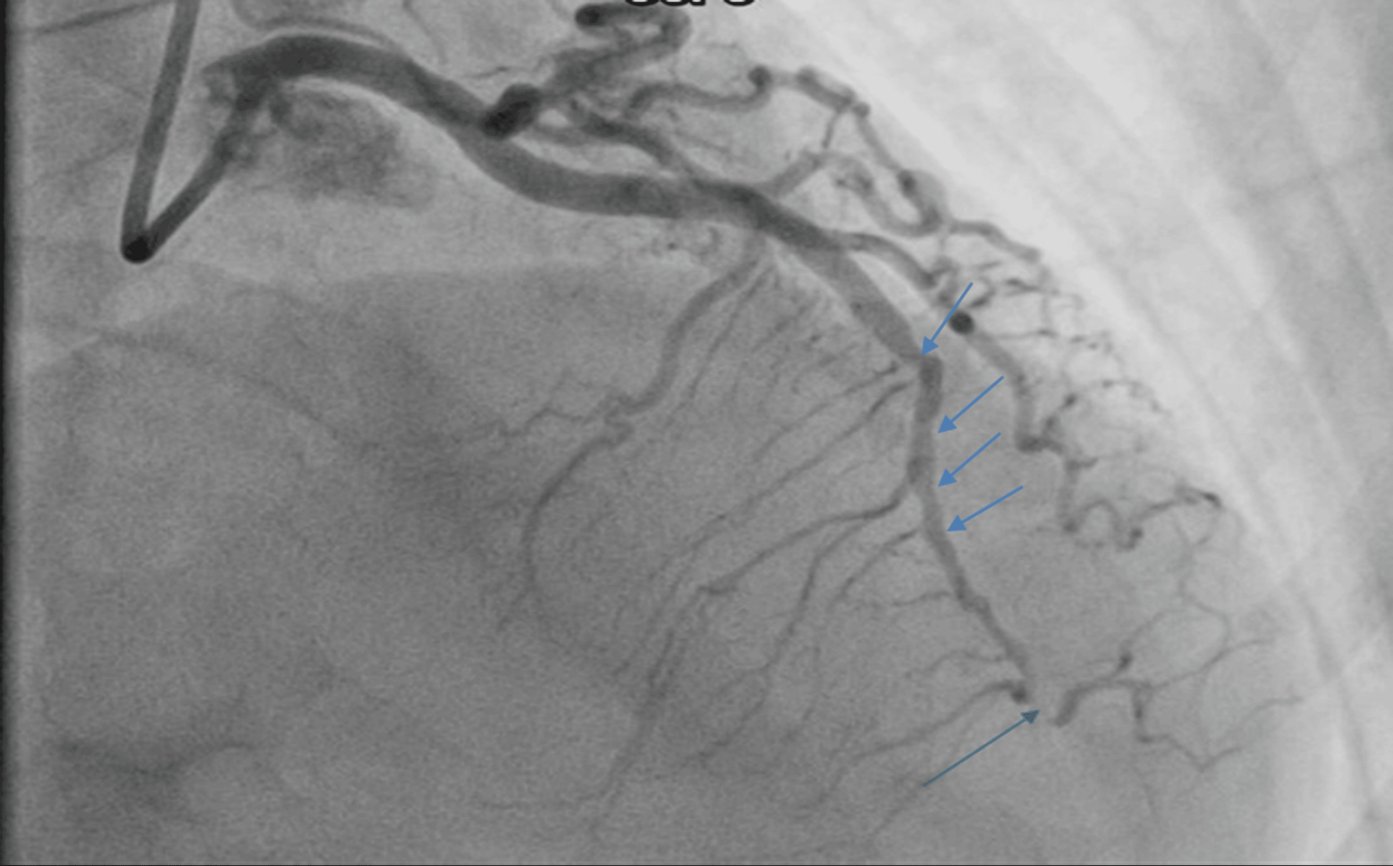
Coronary angiogram (LAO cranial view) demonstrating additional left coronary projections. Multiple arrows indicate the dissected and tapered segments of the mid-to-distal left anterior descending (LAD) artery. These findings are consistent with a type 2 spontaneous coronary artery dissection (SCAD) with preserved distal flow (TIMI 3). LAO: Left anterior oblique.

Coronary angiography revealed significant tortuosity in all coronary arteries (Figures 3-4) with a diffusely irregular distal left anterior descending artery (LAD). There was a sudden change in diameter followed by a long segment of narrowing and eventual subtotal occlusion of the very distal LAD segment, not amenable to percutaneous coronary intervention (PCI) (Figure 5).

Echocardiography ruled out Takotsubo cardiomyopathy and demonstrated a small area of apical hypokinesis with preserved left ventricular ejection fraction (LVEF 55%). The right ventricle (RV) was mildly dilated and hypokinetic. Given the distal location of the lesion and the potential risk of propagating the dissection, intravascular imaging with optical coherence tomography (OCT) or intravascular ultrasound (IVUS) was not pursued.

The patient was initially managed for NSTEMI with standard medical therapy, including dual antiplatelet therapy (aspirin and clopidogrel), anticoagulation (heparin), a beta-blocker (metoprolol), and a statin (atorvastatin). Following coronary angiography, which revealed distal LAD dissection consistent with SCAD, anticoagulation, additional antiplatelet therapy, and statins were discontinued. The patient was subsequently managed medically with low-dose metoprolol succinate 12.5 mg daily and aspirin, as blood pressure was borderline low (100/60 mmHg) with a heart rate of 69 beats per minute. She remained haemodynamically stable, and symptoms resolved with conservative therapy.

The patient’s hospital course, including the chronological sequence of investigations, interventions, and clinical recovery, is summarised in Table 2.

**Table 2:** Concise chronological overview of the patient’s hospital course, from symptom onset to discharge. It summarizes key diagnostic and management milestones, including serial troponin measurements, electrocardiographic changes, angiographic findings, and echocardiographic assessments. The timeline highlights the progression from initial NSTEMI suspicion to angiographically confirmed spontaneous coronary artery dissection (SCAD), emphasizing the rationale for conservative management and subsequent clinical recovery.

Event	Hospital Day	Findings	Key Results / Actions
Symptom onset	Day 0	Substernal chest pain following emotional stress	Presented to ED
Initial ECG / troponin	Day 0	Normal sinus rhythm (NSR), hs-Tn 143 pg/mL	NSTEMI suspicion
Repeat troponin	6 hr	796 pg/mL	Trending upward
Peak troponin	36 hr	9513 pg/mL	Myocardial injury confirmed
ECG evolution	Day 2	ST elevation V3-V6	STEMI pattern observed
Angiography	Day 2	Distal LAD dissection, tortuosity	SCAD demonstrated
Echocardiogram	Day 2	EF 55%, mild RV hypokinesia	No evidence of Takotsubo cardiomyopathy
Discharge	Day 5	Stable, β-blocker/ASA only	Conservative management

Computed tomography angiography (CTA) or magnetic resonance angiography (MRA) of the head, neck, and abdomen was not performed during hospitalisation, as there was no direct clinical indication at the time of admission. However, outpatient vascular imaging is planned to evaluate for FMD or other extracoronary arteriopathies associated with SCAD.

## Discussion

SCAD is an increasingly recognized cause of ACS among young to middle-aged women without traditional atherosclerotic risk factors. It accounts for up to 35% of myocardial infarctions in women under 50 years of age and is strongly associated with pregnancy and the peripartum period. Most pregnancy-associated SCAD (P-SCAD) cases occur within the early postpartum weeks; however, its presentation years later, especially in actively lactating individuals, is exceedingly rare. We present an unusual case of SCAD in a breastfeeding woman three years postpartum, with angiographic evidence of marked coronary tortuosity and distal LAD dissection.

The patient presented with chest pain and dynamic EKG changes consistent with ST-elevation myocardial infarction (STEMI). Coronary angiography demonstrated a focal subtotal occlusion of the distal LAD in the setting of markedly tortuous epicardial coronary arteries. Coronary tortuosity is a common anatomical finding in SCAD and carries important clinical implications. It may reflect an underlying arteriopathy, such as a connective tissue disorder, or result from chronic mechanical stress on the vessel wall. Studies have shown that arterial hypertension in SCAD patients is associated with increased tortuosity and may contribute to the risk of recurrence, highlighting the role of hemodynamic stress in structural vessel changes [7,8].

SCAD is classified angiographically into three types: Type 1 shows a visible intimal flap with contrast entering a false lumen; Type 2, the most common form, presents as diffuse vessel narrowing without a clear flap; and Type 3 mimics focal atherosclerotic stenosis and often requires intravascular imaging for diagnosis [9]. Accurate recognition of these patterns is critical to avoid misdiagnosis and to guide management. In our patient, the angiographic findings, characterized by long, smooth narrowing without evidence of atherosclerotic plaque or embolic source, were consistent with Type 2 SCAD.

The pathophysiology of SCAD is hypothesized to involve hormonal and structural changes that alter vascular integrity. Hormonal fluctuations, particularly reduced estrogen and elevated prolactin levels, may affect collagen synthesis and weaken the arterial wall. During pregnancy, elevated progesterone contributes to medial collagen degradation and elastic fiber disorganization, predisposing to dissection. Heafner et al. proposed a two-phase mechanism involving an initial intimal disruption triggered by hormonal shifts, followed by intramural hemorrhage exacerbated by peripartum coagulopathy [10,11]. Prolonged lactation may perpetuate this hormonally altered vascular state, extending the window of vulnerability to SCAD beyond the immediate postpartum period [10,11]. Further studies are needed to clarify the hormonal influences involved in SCAD. While prolonged lactation may contribute to vascular changes that increase susceptibility to dissection, this relationship remains hypothetical and not yet firmly established.

**Figure 6:**
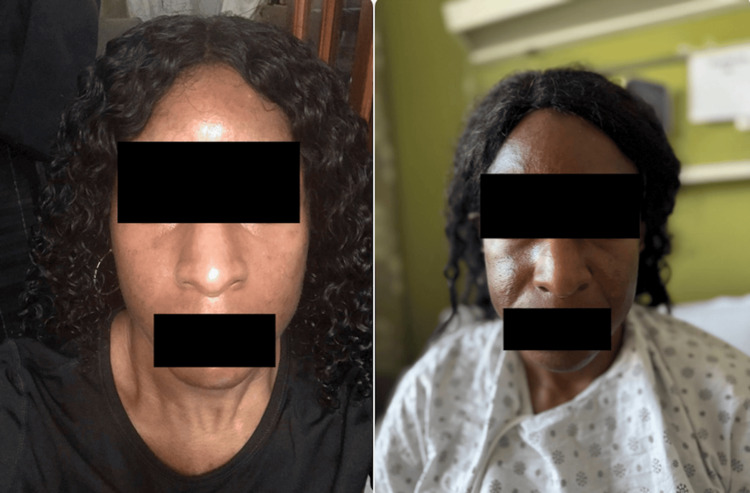
Pre-pregnancy (left) and three years postpartum during hospitalization (right) demonstrating persistent, symmetrical facial melasma, most prominent over the forehead, nasal bridge, and malar regions, likely reflecting prolonged hormonal effects associated with extended lactation.

Considering the patient’s relatively young age, absence of traditional atherosclerotic risk factors, and marked coronary tortuosity observed on angiography, an underlying heritable arteriopathy was considered. Although no genetic testing was performed in this case, several genetic conditions have been associated with increased susceptibility to SCAD. Vascular Ehlers-Danlos syndrome, resulting from mutations in the *COL3A1 gene*, and Loeys-Dietz syndrome, linked to *TGFBR1 and TGFBR2* mutations, are known to predispose individuals to arterial dissections due to connective tissue fragility [12]. Additionally, familial forms of arterial dissection without overt syndromic features have been described, suggesting a broader genetic predisposition in certain cases. While our patient did not have phenotypic features suggestive of a connective tissue disorder, the possibility of an underlying genetic arteriopathy remains a relevant consideration in similar clinical contexts. Figure 6 shows the patient before pregnancy (left) with even skin tone and no hyperpigmentation, and three years postpartum during hospitalization (right) with persistent, symmetrical facial hyperpigmentation (melasma) over the forehead, nasal bridge, and malar regions, suggestive of prolonged hormonal effects from extended lactation. 

SCAD has a recognized risk of recurrence, with studies reporting rates between 10% and 30% over long-term follow-up. In the Canadian SCAD Cohort, the 5-year recurrence rate was approximately 17%, while the Mayo Clinic registry noted up to 30% recurrence. Most repeat events occur in different coronary segments, suggesting a systemic arteriopathy rather than localized vessel weakness. Risk factors include younger age, female sex, FMD, and recurrent physical or emotional stress. Although data are limited, beta-blockers may reduce recurrence risk. Ongoing surveillance, stress avoidance, and patient education remain essential components of long-term care [12,13].

The management of SCAD is nuanced and should be individualized based on clinical stability, extent of dissection, and evidence of ongoing ischemia. This patient was managed conservatively with beta-blockers, antiplatelet therapy, and risk factor modification, consistent with current recommendations for SCAD. SCAD is predominantly treated with conservative medical therapy, as most patients demonstrate spontaneous healing and restoration of normal coronary anatomy within approximately 30 days. This favorable natural history supports the widely accepted “as conservative as possible” approach. Revascularization is generally avoided due to the technical difficulties and risk of propagating the dissection, especially in small-caliber or distal vessels. Invasive strategies are typically reserved for patients with persistent ischemia, hemodynamic compromise, or involvement of high-risk coronary segments such as the proximal left main [12,14]. Serial follow-up imaging is valuable to monitor vessel healing and to screen for underlying arteriopathies such as FMD, which has been reported in more than half of SCAD cases. In our patient, outpatient CT angiography was arranged to evaluate for FMD, along with genetic counseling to explore possible heritable vascular disorders.

## Conclusions

This case highlights a rare presentation of SCAD in a young, actively lactating woman three years postpartum, characterized by angiographic evidence of coronary tortuosity and distal LAD artery involvement. The ECG demonstrated anterolateral MI, and echocardiography revealed unexpected RV dilation and hypokinesia without evidence of pulmonary hypertension or clear signs of RV infarction. These findings, together with the progressive troponin elevation and stable hemodynamic course, support the rationale for conservative management in angiographically confirmed type 2 SCAD. The etiology of the RV dysfunction remains uncertain but may reflect an unrecognized component of SCAD. Hormonal changes associated with pregnancy and prolonged lactation, combined with anatomical factors such as coronary tortuosity, may have contributed to arterial wall vulnerability. This case underscores the importance of recognizing SCAD beyond the immediate postpartum period and considering it in young women presenting with ACS in the absence of atherosclerotic disease.
